# Weakening the Mn–O–Si Interaction via Carbon Intercalation for the Enhanced Catalytic Ozonation of Refractory Pollutants in Environmental Matrices

**DOI:** 10.1021/acsami.4c21068

**Published:** 2025-02-17

**Authors:** Huating Huang, Weiqing Li, Xixi Chen, Zhiming Yang, Minggang Chen, Anhong Zhang, Chun He, Shuanghong Tian

**Affiliations:** † School of Environmental Science & Engineering, Sun Yat-sen University, Guangzhou 510275, P. R. China; ‡ Guangdong Provincial Key Laboratory of Environmental Pollution Control and Remediation, Guangzhou 510275, P. R. China; § Department of Materials Science and Engineering, City University of Hong Kong, Hong Kong 999077, P. R. China; ∥ China National Chemical Southern Construction Investment Co., Ltd., Guangzhou 516000, P. R. China

**Keywords:** catalytic ozonation, metal−support interactions, Si−O−Mn, carbon film, hydrophobicity

## Abstract

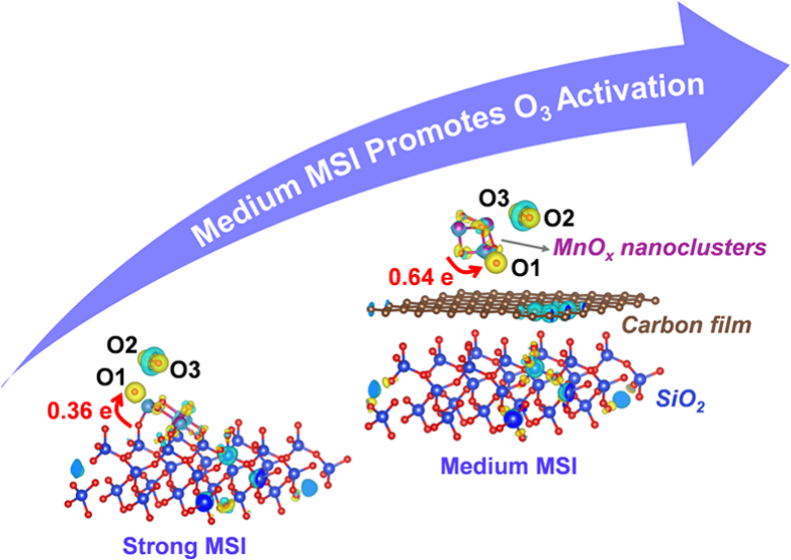

The strong metal–support interaction (MSI) has been widely attributed to enhanced catalytic activity. However, this attribution might be wrong in catalytic ozonation, since MSI that is too strong might impede the activation of electron-poor ozone molecules. Herein, we reported a strategy to subtly modulate the Mn–O–Si interaction by intercalating the carbon film between the silica support and active manganese oxide. When using MnO_*x*_/0.5C/SiO_2_ with the moderate MSI as a catalyst in the catalytic ozonation of refractory paracetamol (PCM), 91.1 ± 2.4% of PCM was removed within 30 min, about 30% higher than that using the catalyst of MnO_*x*_/SiO_2_ with a strong MSI. Moreover, the reaction rate reached 8.01 × 10^–2^ min^–1^, 2.2 and 1.3 times that with MnO_*x*_/SiO_2_ and MnO_*x*_/1C/SiO_2_, respectively. Importantly, further integration of MnO_*x*_/0.5C/SiO_2_ into membrane filtration achieved high rejections of PCM (>94.3%) under various realistic water scenarios during a continuous 12 h operation, demonstrating strong resistance to environmental matrices interference. Experimental and theoretical evidence revealed that the moderate MSI resulted in the high dispersion of active MnO_*x*_ nanoclusters in the size of 2.3–4.4 nm and promoted the adsorption of ozone over MnO_*x*_ and its dissociation into surface *O, ^•^OH, ^•^O_2_^–^, and ^1^O_2_ for decontamination. As a constructive work, this study revealed the significance of MSI in catalytic ozonation and offered a simple regulation method for constructing active interfaces of metal-supported catalysts.

## Introduction

With the acceleration of the industrialization process, more and more toxic and refractory emerging contaminants (ECs) that are resistant to traditional treatment methods have been detected in wastewater^1,2^ and aquatic environments,^3,4^ posing a potential threat to the ecosystem and human health.^5^ Advanced oxidation processes (AOPs) such as catalytic ozonation, Fenton-like reactions, photocatalytic oxidation, persulfate, and peroxymonosulfate oxidation can be applied for the degradation of refractory ECs due to the formation of highly reactive oxygen species (ROS).^6−9^ Among them, catalytic ozonation has received extensive attention because of its mild reaction conditions, simple operation, good adaptability to various pollutants, as well as less secondary pollution.^8,10,11^

In catalytic ozonation, the adsorption and activation of ozone over the catalytic active sites are the core step, which decides the treatment efficiency of this process. As is known, there is a special delocalized π-bond in ozone molecules in which electrons move among three oxygen atoms. On the whole, two terminal oxygen atoms in the ozone molecule are partially negatively charged, with Lewis base characteristics, and ozone is an electron-poor molecule (Figure S1). Therefore, ozone can be adsorbed on the Lewis acid site of the catalyst through Lewis acid–base interaction and can be activated by snatching electrons from the Lewis acid site. It has been demonstrated that in the widely studied MnO_2_-catalyzed ozonation, constructing electron-rich centers around Mn sites and electron-rich oxygen vacancies (O_V_) greatly promoted the degradation of pollutants.^12−14^ Therefore, the surface electronic state of the catalysts, especially the electron-rich state of Lewis acid sites (e.g., metal ions), has a significant impact on catalytic performance.

Nanoclusters with few-atom assemblies have large amounts of exposed and under-coordinated surface atoms, which make them highly active in catalysis. Due to the thermodynamic instability of highly active nanoclusters, they need to be anchored on the supports via metal–support interaction (MSI). In addition to stabilizing active components, MSI could also regulate the electronic state of catalysts and has gained widespread recognition.^15−17^ In essence, the strong metal–support interaction (SMSI) effect is attributed to substantial electronic perturbations caused by electron transfer between the metal and support. Various supports, such as Al_2_O_3_, TiO_2_, and SiO_2_, were applied to supply more stable and active sites for the catalytic ozonation of refractory pollutants. Among them, silica and silica-based zeolites, as one of the most widely used industrial supports, have attracted particular research interest due to their stability, low cost, good dispersibility, and abundant anchoring site. For example, SiO_2_ spheres,^18^ SBA-15^19,20^ and Al-containing zeolite,^21^ have been used as carriers of ozone catalysts. All of these investigations demonstrated that the silica-based supports improved the catalytic performance. The improvement was exclusively attributed to the high dispersion of active components or the strong metal–support interaction (SMSI) effect between active metal oxides and silica-based supports. Due to the SMSI induced from the chemically active surface, such as the presence of hydroxyls over the oxides, Si–O–M (metal) bonds undoubtedly favored the stable dispersion of active metal components and maximized their exposure to the reactants, thus promoting the reaction. Could SMSI really work positively in catalytic ozonation? In theory, Si, with strong electron-attraction ability in silica-based support, seriously attracts electrons from metal when Si–O–M bonds are formed.^19,22−24^ As mentioned above, the electron-poor state of Lewis acid sites is detrimental to the activation of electron-poor ozone. In practice, silica-based supports were reported to show increased catalytic performance but exhibit worse than other supports of Al_2_O_3_ and TiO_2_.^25,26^ Based on the above facts and the design of other active structures,^27−30^ it is clear that continued enhancement of the MSI without restriction will lead to a further transfer of electrons from metal to Si via the Si–O–M bond, which inevitably results in an elevated valence state of the metal and impedes electron transfer from Lewis acid sites (metal) to ozone. In this context, the influence of MSI on catalytic properties and mechanisms and the strategies for regulating MSI in catalytic ozonation must be reconsidered.

In this work, the widely investigated manganese oxide (MnO_*x*_) was employed as the model active components and SiO_2_ nanoparticle as the model silica support to investigate the MSI for catalytic ozonation of refractory pollutants in environmental matrices. Considering that carbon materials are known to exhibit weak MSI,^31,32^ we introduced a carbon film on the surface of SiO_2_ via a simple glucose-assisted thermal treatment method to regulate the MSI between MnO_*x*_ and SiO_2_. Particularly, both experiments and density functional theory (DFT) calculations were conducted to reveal the role of MSI at an atomic level in catalytic ozonation. As a constructive work, the objective is to disclose an easy regulation method for constructing active interfaces of metal-supported catalysts and to illustrate the significance of regulating MSI of catalysts in catalytic ozonation.

## Experimental Section

### Materials

Silicon dioxide (SiO_2_) nanoparticles and d-(+)-glucose were purchased from Shanghai Macklin Biochemical Co., Ltd. Manganese(II) acetate tetrahydrate, 5,5-dimethyl-1-pyrroline N-oxide (DMPO), dimethyl sulfoxide (DMSO), 2,2,6,6-tetramethylpiperidine (TEMP), benzoquinone (pBQ), and paracetamol (PCM) were obtained from Aladdin Chemistry Co., Ltd. Tert-butyl alcohol (TBA) was bought from Tianjin Baishi Chemical Reagent Co., Ltd. (China). All chemicals were of analytical grade and used without further purification. The deionized water used in the experiments was obtained from a Millipore system.

### Preparation of Catalysts

#### Preparation of *x*C/SiO_2_

*x*C/SiO_2_ were synthesized by the reported method.^31^ One gram of SiO_2_ and a certain amount of glucose aqueous solution were mixed and dried. The dry mixture was transferred to a tube furnace for heat treatment at 650 °C for 4 h in an argon atmosphere. After cooling, carbon-coated SiO_2_ was obtained. The samples were designated as *x*C/SiO_2_ (*x* = 0, 0.5, and 1), where *x* is the mass ratio of glucose/SiO_2_.

#### Preparation of MnO_*x*_/*x*C/SiO_2_

A certain mass of Mn(CH_3_COO)_2_·4H_2_O was dissolved in 3.8 mL of deionized water, and then the solution was dropped into 1 g of *x*C/SiO_2_ powder. After thorough grinding and mixing, the powder was dried at 80 °C for 6 h, followed by heat treatment at 650 °C for 4 h in an argon atmosphere. The sample was named MnO_*x*_/*x*C/SiO_2_. The theoretical loading of Mn was 5.0 wt %.

#### Characterization and Analysis Methods

The characterization of the prepared catalysts and the analysis method employed are described in Text S1.

#### Catalytic Ozonation Procedures

Tests were conducted in both batch reactors and a continuous-flow membrane reactor. The details are described in Text S2.

#### Computational Methods

Density functional theory calculations were performed following the projector-augmented plane-wave method as implemented in the Vienna ab initio simulation package (VASP).^33−35^ The details are described in Text S3.

## Results and Discussion

### Controllable Preparation of MnO_*x*_/xC/SiO_2_

**Figure 1:**
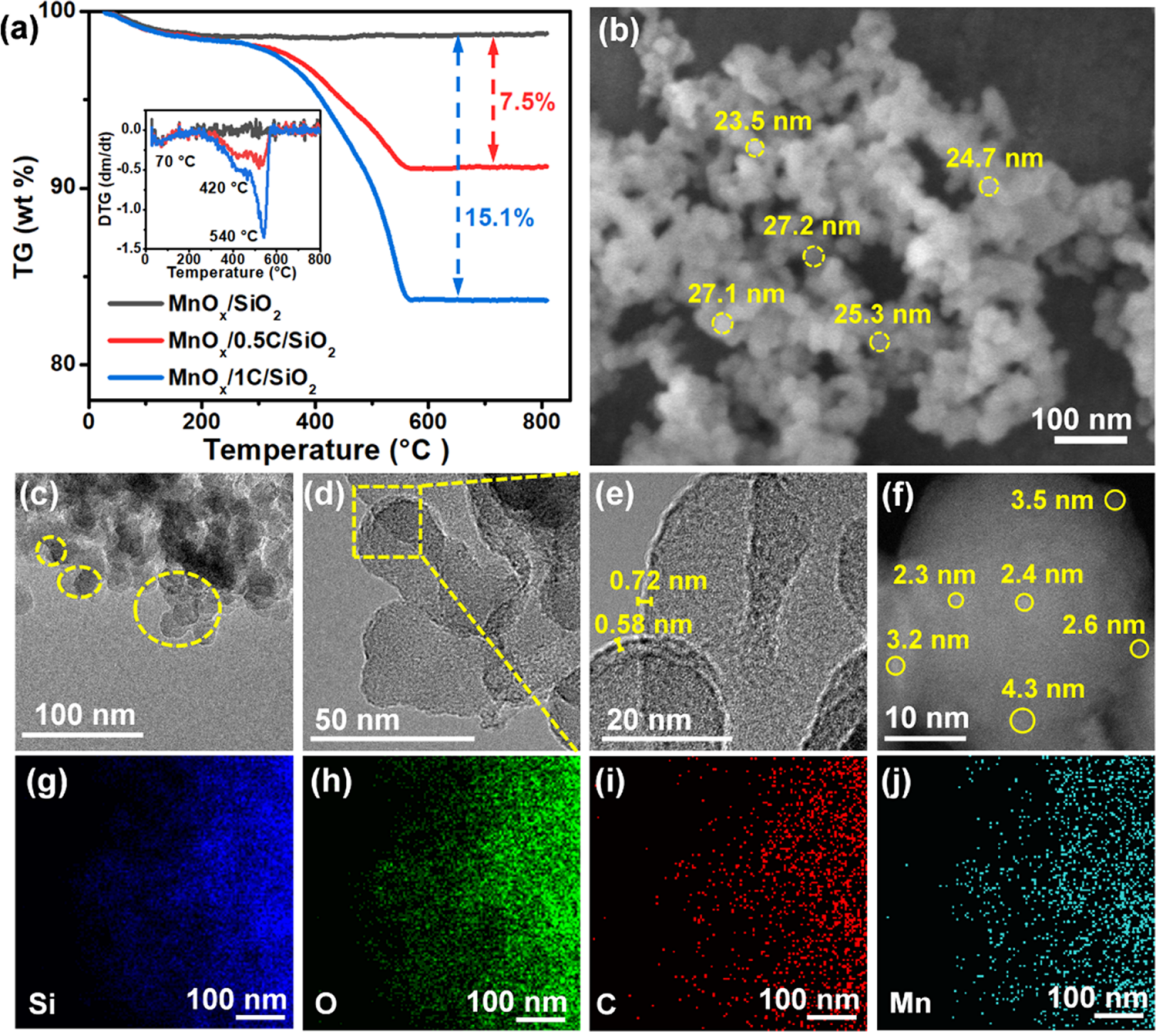
(a) TGA results of MnO_*x*_/*x*C/SiO_2_, (b) SEM images, (c–e) TEM images, (f) AC-HAADF-STEM images, and (g–j) EDS elemental mapping images of MnO_*x*_/0.5C/SiO_2_.

The carbon-coated SiO_2_ (*x*C/SiO_2_) support was simply prepared by impregnation of various amounts of glucose and subsequent thermal treatment under an argon atmosphere. The content of the C element was confirmed by thermogravimetric analysis (TGA). As shown in Figure S2, the mass loss occurring from 200 to 600 °C was attributed to the decomposition of the carbon film in air.^18^ By calculation, 0.5C/SiO_2_ and 1C/SiO_2_ had 9.2 and 16.4% carbon, respectively. Furthermore, the MnO_*x*_/*x*C/SiO_2_ catalysts were obtained by loading 5.0 wt % Mn on *x*C/SiO_2_. The mass losses of MnO_*x*_/SiO_2_, MnO_*x*_/0.5C/SiO_2_, and MnO_*x*_/1C/SiO_2_ at 200–600 °C were 0, 7.5, and 15.1% (Figure 1a and Table S1), respectively. The latter two seemed to be slightly lower than those of the *x*C/SiO_2_ supports. It is reasonable when considering the mass of MnO_*x*_. The obtained MnO_*x*_/0.5C/SiO_2_ had a morphology of nanospheres, about 25 nm in diameter, which was very similar to that of the bare SiO_2_ support (Figure 1b,c). It can be seen from Figures 1d,e and S3 that the silica spheres were covered by amorphous carbon films with a thickness of about 0.5–1.0 nm. Moreover, no obvious MnO_*x*_ particles were observed even in the HRTEM images of MnO_*x*_/0.5C/SiO_2_, but high brightness and tiny nanoclusters with a size of 2.3–4.4 nm on the surface of the MnO_*x*_/0.5C/SiO_2_ catalyst were observed in the AC-HAADF-STEM image (Figure 1f), indicating the high dispersion of small-size MnO_*x*_ nanoclusters on the surface of the 0.5C/SiO_2_ support. The EDS elemental mapping (Figure 1g–j) displayed the uniform distribution of the C and Mn elements on the surface of SiO_2_. The combined results confirmed that MnO_*x*_ nanoclusters supported on carbon-coated silica catalyst (MnO_*x*_/0.5C/SiO_2_) were successfully prepared.

**Figure 2:**
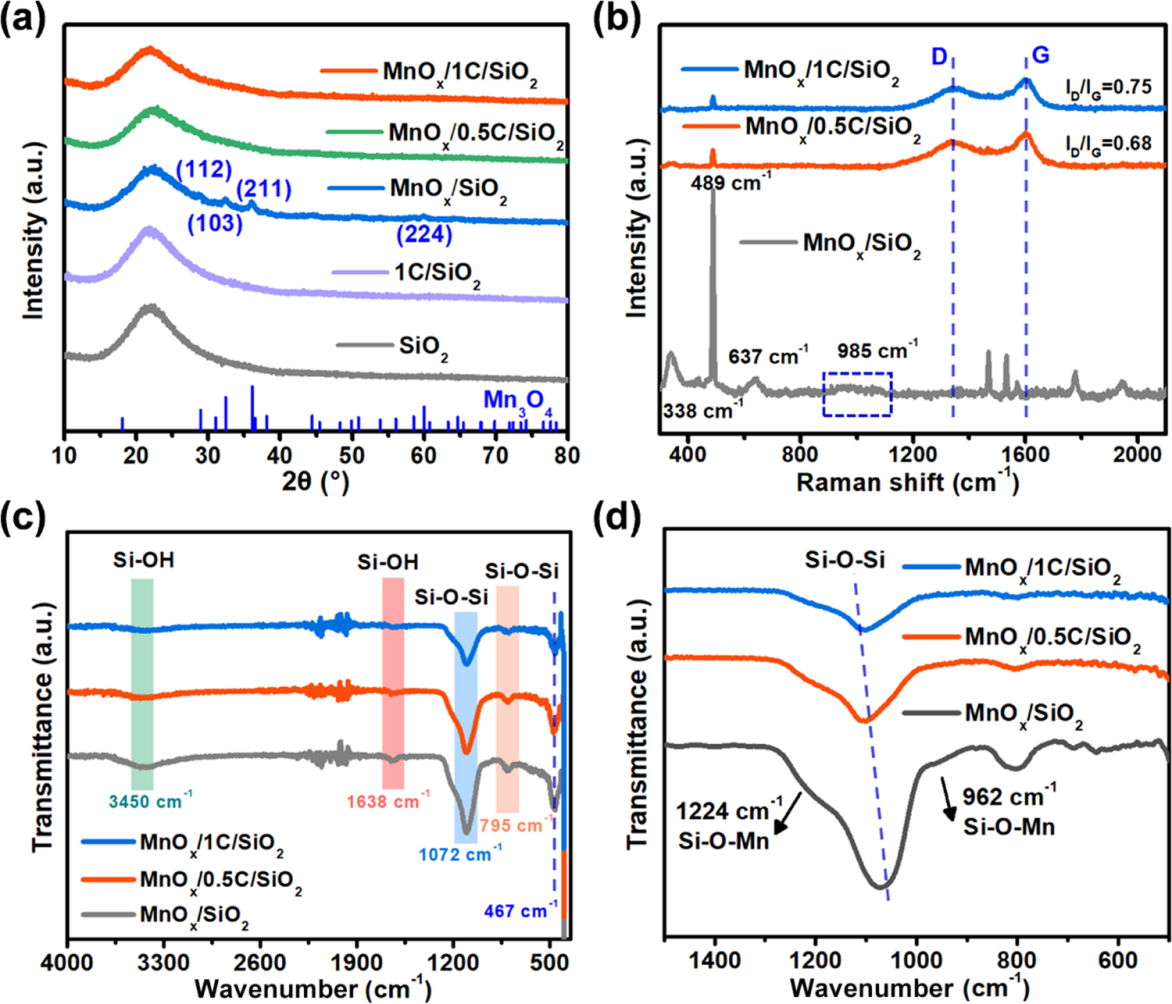
(a) XRD patterns, (b) Raman spectra, and (c, d) FTIR spectra of the catalysts.

The XRD diffractogram of all samples displayed a broad diffraction peak corresponding to amorphous SiO_2_ at 22° (Figure 2a). No peaks of graphene were found, indicating that the carbon films on all of the samples were amorphous. Instead, MnO_*x*_/SiO_2_ without C-modification displayed four diffraction peaks at 28.93, 32.39, 36.10, and 59.94°, corresponding to the (112), (103), (211), and (224) planes of Mn_3_O_4_ (JCPDS no. 75-1560), indicating that large Mn_3_O_4_ particles accumulated during the preparation possibly due to the limited Si–OH groups on the SiO_2_ carrier for anchoring Mn species.^19^ In contrast, the diffraction of Mn_3_O_4_ disappeared on all of the C-modified catalysts, implying the small size and/or high dispersion of MnO_*x*_ on *x*C/SiO_2_. The XRD results indicated that the introduction of carbon hindered the agglomeration, migration, and sintering of MnO_*x*_, which favored the exposure of the catalytic active sites.^31^

To further understand the role of carbon modification, both Raman and FTIR spectroscopies were used to investigate the MSI between MnO_*x*_ and SiO_2_. As displayed in the Raman spectra of Figure 2b, the strong and sharp characteristic peaks located at 338 and 489 cm^–1^ in MnO_*x*_/SiO_2_ could be assigned to the bending vibration of the O–Mn–O, while that at 637 cm^–1^ was related to the breathing vibration of the Mn–O–Mn bond in the [MnO_6_] octahedral.^36,37^ This further confirmed the deposition of large MnO_*x*_ particles on SiO_2_. Furthermore, a weak and wide peak at 985 cm^–1^ was observed, which was attributed to the asymmetric tensile vibrations of Si–O–Mn in MnO_*x*_/SiO_2_.^38^ Compared to MnO_*x*_/SiO_2_, MnO_*x*_/*x*C/SiO_2_ showed significantly different Raman characteristic peaks. The disappearance of peaks at 338 and 637 cm^–1^ indicated that the introduction of C weakened the Mn–O–Mn bond during the transition of MnO_*x*_ from crystalline to amorphous state.^39^ Meanwhile, the characteristic peak of the Si–O–Mn bond at 985 cm^–1^ disappeared in MnO_*x*_/*x*C/SiO_2_. This implied that the active MnO_*x*_ was anchored on the carbon film rather than directly on SiO_2_. This change demonstrated that the MSI between the active component and the support could be tuned by carbon modification. It was noticed that the characteristic peak attributed to carbon occurred in the Raman spectrum of MnO_*x*_/*x*C/SiO_2_. The intensity ratio of the D band at 1345 cm^–1^ and the G band at 1600 cm^–1^ (*I*_D_/*I*_G_) is commonly used to characterize the graphitization level of carbon, and a lower *I*_D_/*I*_G_ value means a higher graphitization level.^40^ As fitting from the Raman spectra, increasing the glucose dosage from 0.5 to 1 increased the *I*_D_/*I*_G_ value from 0.68 to 0.75, indicating that the excessive glucose addition was not conducive to the graphitization of MnO_*x*_/*x*C/SiO_2_. However, the *I*_D_/*I*_G_ value of MnO_*x*_/0.5C/SiO_2_ (0.68) was much smaller than that of many carbon materials prepared at the same temperature,^41−43^ possibly because SiO_2_ could be a potential graphitization catalyst in the carbonization process.^44^ In a word, the higher graphitization level of the catalysts can be obtained by controlling the ratio of glucose/SiO_2_, which favors the electron transfer in the catalytic ozonation process.^45^

The FTIR spectra (Figure 2c,d) further revealed the role of carbon modification. As shown in Figure 2c, the bands at 467 cm^–1^ were related to the stretching vibration of the Mn–O bond,^46^ the intensities of which followed the order of MnO_*x*_/SiO_2_ > MnO_*x*_/0.5C/SiO_2_ > MnO_*x*_/1C/SiO_2_. It could be explained by two reasons: (i) the intervention of carbon hindered the electron transfer from Mn to Si in the Si–O–Mn bond, thus weakening the Mn–O bond and (ii) the weakening and hindering effect of carbon on the infrared signal. The bands at 795 and 1072 cm^–1^ were assigned to the bending vibration and asymmetric stretching vibration of the Si–O–Si bonds, respectively. As shown in Figure 2d, the peaks of the Si–O–Si bonds red-shifted to lower wavenumbers, indicating that the interaction between MnO_*x*_ and SiO_2_ was weakened.^47^ Meanwhile, the peaks of the Si–O–Mn bonds located at 962 and 1224 cm^–1^ were also weakened after the carbon introduction,^48,49^ further indicating the weakening effect of the MSI in MnO_*x*_/*x*C/SiO_2_. The Raman and FTIR results collectively demonstrated that the introduction of carbon weakened the interactions between the SiO_2_ support and the active MnO_*x*_ component.

In addition, the bands at about 3450 and 1638 cm^–1^ in Figure 2c were attributed to the surface Si–OH groups and adsorbed water molecules. The increase in carbon weakened the intensities of these two bands in MnO_*x*_/*x*C/SiO_2_. Thus, it was speculated that the increase of carbon led to the increase of hydrophobicity of the MnO_*x*_/*x*C/SiO_2_ catalysts, resulting in the reduced adsorption of water molecules on the catalyst surface.^50^ The enhanced hydrophobicity might favor the adsorption of ozone and organic pollutants over the MnO_*x*_/*x*C/SiO_2_ catalysts.

**Figure 3:**
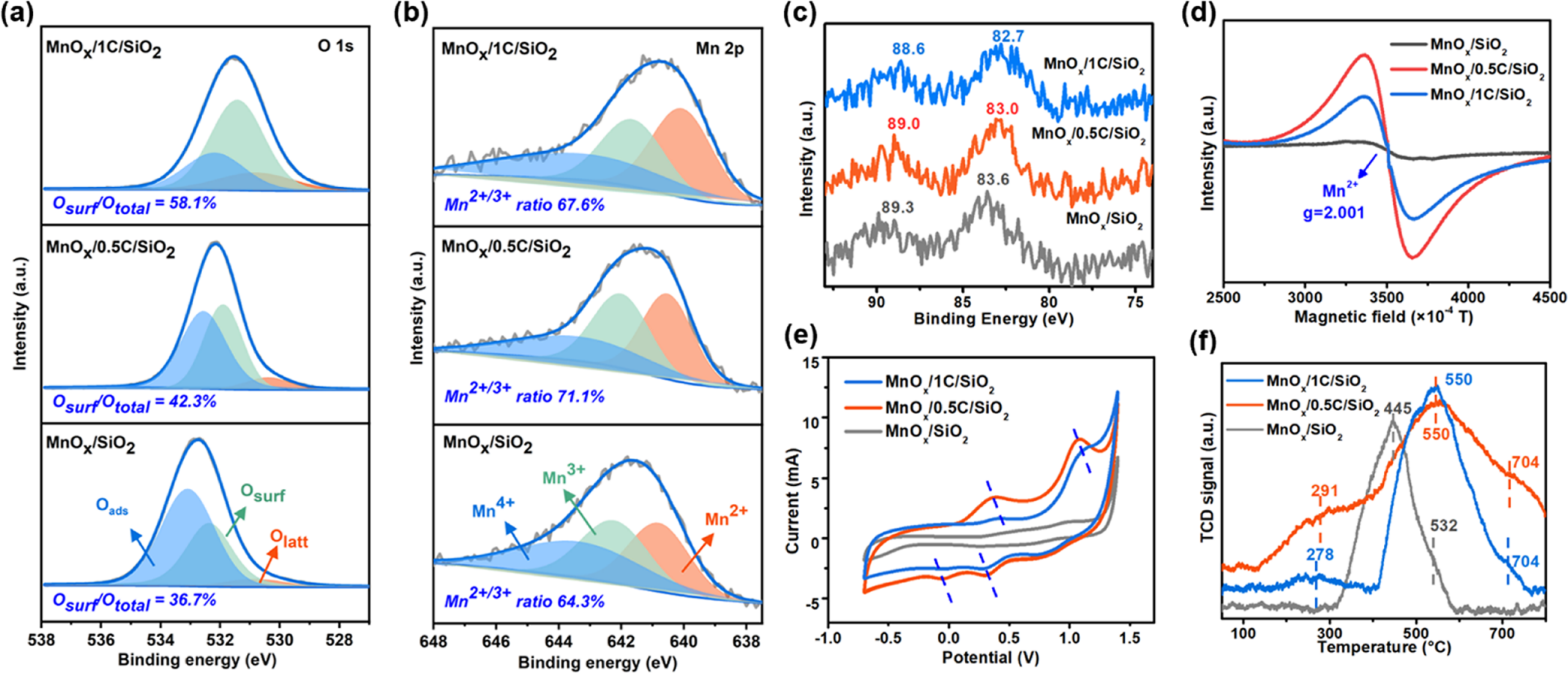
XPS spectra of (a) O 1s, (b) Mn 2p, and (c) Mn 3s. (d) ESR patterns, (e) CV curves, and (f) H_2_-TPR of MnO_*x*_/SiO_2_, MnO_*x*_/0.5C/SiO_2_, and MnO_*x*_/1C/SiO_2_.

XPS spectroscopy was used to study the surface chemical composition and chemical state of MnO_*x*_/*x*C/SiO_2_ with different MSI strengths. As presented in Figure 3a, the XPS spectrum of O 1s could be deconvoluted into three peaks located at about 530.3, 531.9, and 533.2 eV, assigned to the lattice oxygen (O_latt_), surface-adsorbed oxygen (O_surf_), and the oxygen atoms on surface-adsorbed water (O_ads_), respectively.^51^ The area ratio of [O_ads_]/[O_total_] followed the order MnO_*x*_/SiO_2_ (58.5%) > MnO_*x*_/0.5C/SiO_2_ (51.2%) > MnO_*x*_/1C/SiO_2_ (26.7%) (Table S1). Obviously, the concentration of O_ads_ decreased with C modification, indicating the enhancement of the hydrophobicity of the MnO_*x*_/*x*C/SiO_2_ catalysts, which was in good agreement with the results of FTIR (Figure 2c) and contact angle tests (Figure S4). O_surf_, generated by the adsorption of gaseous O_2_ into oxygen vacancies (OVs), plays a more important role in catalytic activity because of its higher mobility than O_latt_.^26^ The area ratio of [O_surf_]/[O_total_] followed the order of MnO_*x*_/1C/SiO_2_ (58.1%) > MnO_*x*_/0.5C/SiO_2_ (42.3%) > MnO_*x*_/SiO_2_ (36.7%), suggesting that the weakness of the MSI possibly facilitated the formation of OVs.

Mn 2p_3/2_ signals of MnO_*x*_/SiO_2_, MnO_*x*_/0.5C/SiO_2_, and MnO_*x*_/1C/SiO_2_ are displayed in Figure 3b. The spectrum could be divided into three peaks located at 640.5, 642.0, and 643.0 eV, which were classified as the binding energies of Mn^2+^, Mn^3+^, and Mn^4+^, respectively.^26,52^ The area ratio of [Mn^2+^]/[Mn_total_] obeyed the order of MnO_*x*_/1C/SiO_2_ (37.6%) > MnO_*x*_/0.5C/SiO_2_ (36.1%) > MnO_*x*_/SiO_2_ (31.9%) (Table S1), consistent with the order of [O_surf_]/[O_total_] ratio reflecting the OVs content. The ratio of ([Mn^2+^]+[Mn^3+^])/[Mn_total_] followed the order MnO_*x*_/0.5C/SiO_2_ (71.1%) > MnO_*x*_/1C/SiO_2_ (67.6%) > MnO_*x*_/SiO_2_ (64.3%), indicating that there were more abundant low-valent Mn^2+^ and Mn^3+^ in MnO_*x*_/0.5C/SiO_2_. Meanwhile, the average oxidation state (AOS) of Mn was quantified by the equation “AOS = 8.956 – 1.126Δ*E*”.^53^ The binding energy difference (Δ*E*) between the two Mn 3s peaks is shown in Figure 3c. The lowest AOS value of MnO_*x*_/0.5C/SiO_2_ (2.20) also suggested that MnO_*x*_/0.5C/SiO_2_ contained a higher content of low-valent Mn than MnO_*x*_/1C/SiO_2_ and MnO_*x*_/SiO_2_. These results indicated that the carbon modification facilitated the generation of low-valent Mn in MnO_*x*_/0.5C/SiO_2_, which might be due to the reduced charge transfer from Mn to SiO_2_ support.^30^ The weakened MSI, the increased hydrophobicity, conductivity, and OV concentration, and the low-valent states of Mn species might favor the enrichment and activation of ozone on the surface of MnO_*x*_/0.5C/SiO_2_ and MnO_*x*_/1C/SiO_2_.

ESR spectroscopy was further used to investigate the oxidation state of the Mn ions in the catalysts. As displayed in Figure 3d, the ESR signal located at *g* = 2.001 was obvious, indicating the presence of Mn^2+^ in the three catalysts.^54^ However, the ESR signal did not exhibit the typical sextet characteristics of Mn^2+^, and none of the samples exhibited Mn^4+^ signs with a *g* value of 1.994, possibly due to the electrostatic or dipolar interaction among various Mn ions with mixed valence states.^55^ In addition, the ESR signal intensity related to Mn^2+^ increased with carbon in MnO_*x*_/*x*C/SiO_2_, consistent with the XPS results.

The redox capacity of the catalysts with different MSI was investigated by the electrochemical CV method. As shown in Figure 3e, the anodic current oxidation peaks corresponded to the oxidation process from Mn^2+^ → Mn^3+^ to Mn^3+^ → Mn^4+^; conversely, the cathodic current reduction peaks were related to the reduction process from Mn^4+^ → Mn^3+^ to Mn^3+^ → Mn^2+^.^56^ MnO_*x*_/0.5C/SiO_2_ owned the oxidation peaks at lower voltage and reduction peaks at higher voltage, indicating that the redox cycle of Mn species in MnO_*x*_/0.5C/SiO_2_ was easier than that in MnO_*x*_/SiO_2_ and MnO_*x*_/1C/SiO_2_.^57^ Furthermore, the current intensities of both oxidation and reduction curves of MnO_*x*_/0.5C/SiO_2_ were much higher than those of MnO_*x*_/SiO_2_ and MnO_*x*_/1C/SiO_2_, implying the highest surface electrons in Mn species of MnO_*x*_/0.5C/SiO_2_.^56^ Therefore, it can be proposed that MnO_*x*_/0.5C/SiO_2_ with weak MSI owned the best redox cycle ability, which is essential for sustainable catalytic ozonation.^56,58^

The redox property of MnO_*x*_ in catalytic ozonation is mainly determined by their reducibility (e.g., Mn^4+^ → Mn^3+^ and Mn^3+^ → Mn^2+^), since the oxidation step is relatively facile in the oxidation environment. H_2_-TPR was thus employed to evaluate the reducibility of the catalysts. As depicted in Figure 3f, both MnO_*x*_/0.5C/SiO_2_ and MnO_*x*_/1C/SiO_2_ contained three peaks at ∼291, 550, and 704 °C, corresponding to the continuous reduction of MnO_2_ → Mn_2_O_3_ → Mn_3_O_4_ → MnO via H_2_ consumption, respectively.^26,59^ In contrast, MnO_*x*_/SiO_2_ exhibited only one overlapped reduction peak located at 445 °C, mainly caused by the reduction of Mn_3_O_4_ → MnO.^49^ It is reported that peaks below 300 °C are due to the reduction of active surface oxygen species.^59^ Apparently, both MnO_*x*_/1C/SiO_2_ (278 °C) and MnO_*x*_/0.5C/SiO_2_ (291 °C) owned lower temperature of the first reduction peak than that of MnO_*x*_/SiO_2_ (445 °C), indicating that MnO_*x*_/1C/SiO_2_ and MnO_*x*_/0.5C/SiO_2_ were more easily reduced by H_2_ and had more surface oxygen species, in good accordance with the XPS O 1s results. Moreover, the total normalized H_2_ consumption followed the sequence of MnO_*x*_/0.5C/SiO_2_ (6.47 cm^3^/g) > MnO_*x*_/1C/SiO_2_ (5.89 cm^3^/g) > MnO_*x*_/SiO_2_ (4.55 cm^3^/g), emphasizing that MnO_*x*_/0.5C/SiO_2_ with poor MSI had the highest content of reducible species.^60^ Therefore, the weak MSI induced by carbon modification in MnO_*x*_/0.5C/SiO_2_ greatly enhanced the reducibility and mobility of surface oxygen species, which would favor the redox cycle and the formation and transformation of ROS during the catalytic ozonation process.

### Catalytic Performance of MnO_*x*_/xC/SiO_2_

**Figure 4:**
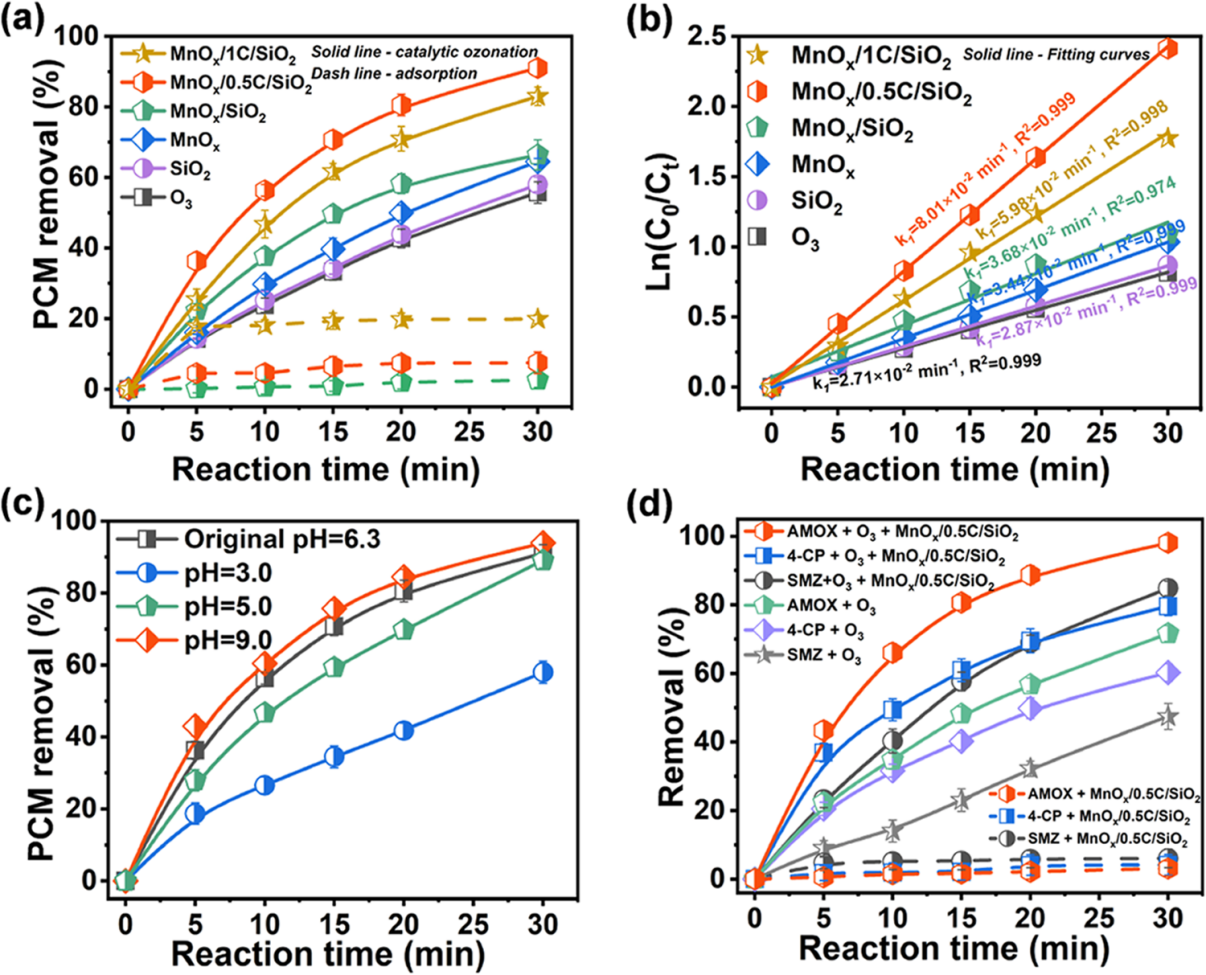
(a) PCM removal and (b) pseudo-first-order kinetics fitting for PCM removal in catalytic ozonation with catalysts. (c) PCM removal at different initial pH values and (d) removal of various pollutants in MnO_*x*_/0.5C/SiO_2_-mediated catalytic ozonation.

Herein, the catalytic activities of various MnO_*x*_/*x*C/SiO_2_ catalysts were investigated by the degradation of PCM. The adsorption capacities of PCM by the three catalysts within 30 min followed the order of MnO_*x*_/1C/SiO_2_ (19.8 ± 1.3%) > MnO_*x*_/0.5C/SiO_2_ (7.5 ± 3.1%) > MnO_*x*_/SiO_2_ (2.6 ± 2.1%) (Figure 4a). Similarly, the adsorption capacities of PCM by the three supports within 30 min also followed the same order of 1C/SiO_2_ (16.5 ± 1.6%) > MnO_*x*_/0.5C/SiO_2_ (8.6 ± 1.6%) > MnO_*x*_/SiO_2_ (1.2 ± 1.1%) (Figure S5). It indicated that the increase of carbon on the SiO_2_ support significantly improved the PCM adsorption. It was probably because carbon modification increased the hydrophobicity and then favored the accumulation of organic pollutants on their surface.

Compared with adsorption, oxidation can more efficiently remove PCM from the system (Figure 4a). In O_3_ alone, 55.7 ± 3.1% of PCM was removed within 30 min. After the introduction of MnO_*x*_/0.5C/SiO_2_ and MnO_*x*_/1C/SiO_2_, the removal of PCM reached 91.1 ± 2.4 and 83.0 ± 2.6%, respectively, much higher than those of O_3_-alone and MnO_*x*_/SiO_2_-catalyzed ozonation. The catalytic activity in terms of PCM degradation by catalytic ozonation followed the order MnO_*x*_/0.5C/SiO_2_ > MnO_*x*_/1C/SiO_2_ > MnO_*x*_/SiO_2_. In particular, MnO_*x*_/0.5C/SiO_2_ possessed the highest first-order rate constant (*k*_1_) of 8.01 × 10^–2^ min^–1^ (Figure 4b), which was 3.0, 2.2, and 1.3 times that of single, MnO_*x*_/SiO_2_, and MnO_*x*_/1C/SiO_2_ ozonation, respectively. As discussed above, the introduction of carbon film weakened the MSI between MnO_*x*_ and SiO_2_ and thus increased the low-valent Mn abundance and redox properties of MnO_*x*_/*x*C/SiO_2_, which greatly facilitated the adsorption and activation of ozone. Moreover, the carbon modification also enhanced the conductivity and hydrophobicity of MnO_*x*_/*x*C/SiO_2_, which promoted the redox cycle and pollutant accumulation around the catalyst. All of these characteristics derived from carbon modification contributed to the high catalytic activity of MnO_*x*_/*x*C/SiO_2_.

The catalytic performance of MnO_*x*_/0.5C/SiO_2_ was further investigated under various initial pH values (3.0–9.0), different target organic pollutants, and natural water matrix. As shown in Figure 4c, MnO_*x*_/0.5C/SiO_2_ exhibited excellent PCM removal efficiency (>89.0 ± 2.1%) over the broad pH range of 5.0–9.0, suggesting its wide pH tolerance. MnO_*x*_/0.5C/SiO_2_ also showed impressive catalytic activity for widely used recalcitrant pollutants that cause environmental and public health concerns, including amoxicillin (AMOX), 4-chlorophenol (4-CP), and sulfamethoxazole (SMZ) (Figure 4d). In the presence of O_3_, MnO_*x*_/0.5C/SiO_2_ exhibited the high removal of AMOX, 4-CP, and SMZ, 26.5, 19.2, and 37.4% higher than those in single ozonation systems, respectively. Furthermore, the practical application feasibility of MnO_*x*_/0.5C/SiO_2_ was investigated through the elimination of PCM in different natural water matrices (deionized water, tap water, surface water, and simulated aquaculture wastewater). The detailed water quality parameters (e.g., pH, COD_Mn_, TOC, UV_254_, turbidity, and DO) are summarized in Table S2. As shown in Figure S6, the degradation of PCM was only slightly inhibited in these realistic water matrices when compared to that in deionized water. This slight inhibition might be attributed to the competition with natural organic matter in the natural water matrices. The catalytic stability of MnO_*x*_/0.5C/SiO_2_ was evaluated with six cyclic experiments. The used catalyst was collected by centrifugation and then washed and dried for the next cycle of catalysis without the addition of fresh catalysts. As shown in Figure S7, the PCM removal reached 91.3% within 30 min after six consecutive experiments, demonstrating the excellent reusability performance of MnO_*x*_/0.5C/SiO_2_ catalyst in the robust oxidation environment.

**Figure 5:**
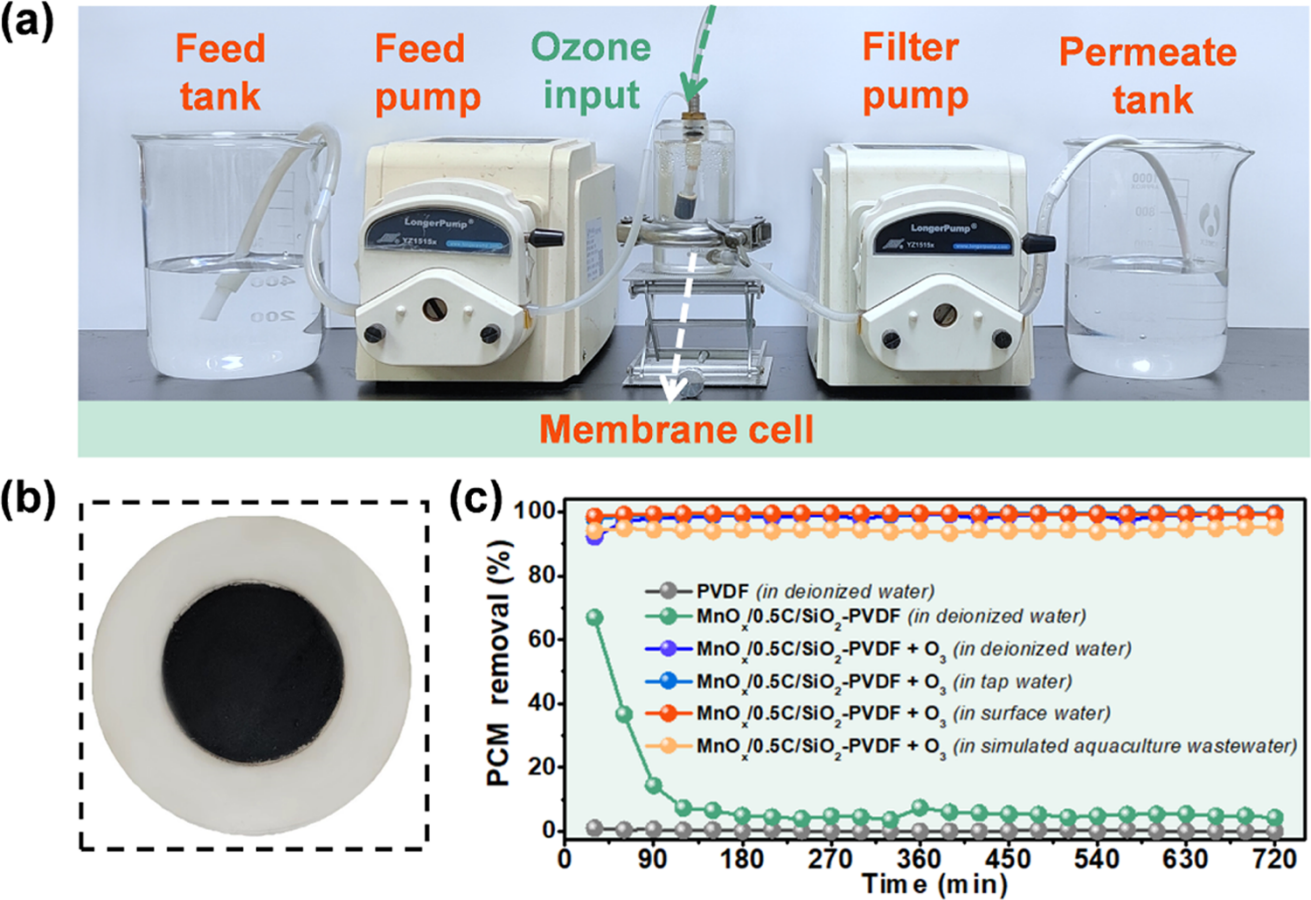
(a) Photograph of the continuous-flow device for membrane filtration. (b) Photograph of the prepared MnO_*x*_/0.5C/SiO_2_/PVDF membrane. (c) PCM removal efficiency using the MnO_*x*_/0.5C/SiO_2_/PVDF membrane for real water matrix.

The results encouraged us to further investigate device integration for long-term water purification. The catalytic membrane filtration process has been studied as a potential strategy for water decontamination due to its high efficiency and sustainability compared to dispersed catalyst suspensions. In this study, a homemade filtration device was assembled for continuous catalytic filtration experiments with ozone supply (see Figure 5a). The MnO_*x*_/0.5C/SiO_2_–PVDF membrane with an effective area of 9.6 cm^2^ was fabricated by vacuum-assisted filtering MnO_*x*_/0.5C/SiO_2_ suspensions on polyvinylidene fluoride (PVDF) substrates (Figure 5b). As shown in Figure 5c, the pristine PVDF membrane and MnO_*x*_/0.5C/SiO_2_/PVDF membrane contributed only 0 and 4.3% rejection of PCM during the 12 h continuous run, respectively. With the addition of O_3_, on average, 99.4, 99.4, 99.4, and 94.3% of PCM were removed from deionized water, tap water, surface water, and simulated aquaculture wastewater, respectively. This suggested the excellent catalytic activity and stability of MnO_*x*_/0.5C/SiO_2_/PVDF toward O_3_ activation. Collectively, the superior reactivity, stability, and high pH adaptability of the MnO_*x*_/0.5C/SiO_2_ catalyst in degrading refractory organic pollutants demonstrated its enormous potential for practical application in water and wastewater decontamination.

### Catalytic Mechanism

#### ROS Identification

**Figure 6:**
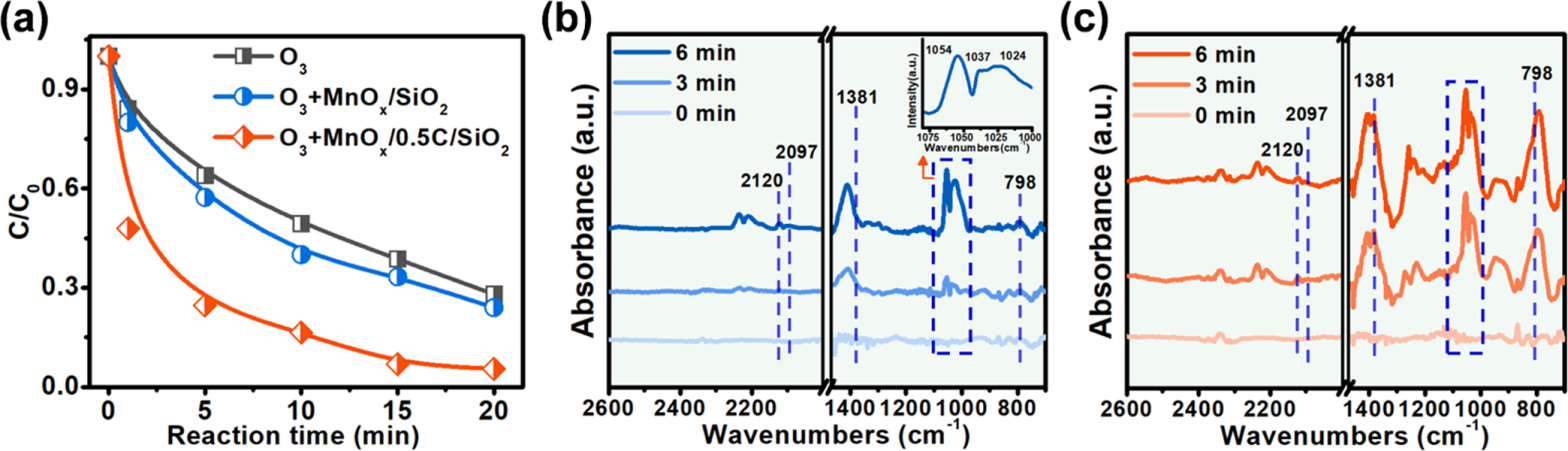
(a) Evolution of the ozone concentration over time in ozonation and catalytic ozonation. In situ DRIFTS spectra of O_3_ adsorption and decomposition over (b) MnO_*x*_/SiO_2_ and (c) MnO_*x*_/0.5C/SiO_2_.

In general, the catalytic ozonation performance is closely related to the decomposition and conversion of ozone into ROS-like singlet oxygen (^1^O_2_), superoxide radical (O_2_^•–^), and hydroxyl radical (^•^OH), resulting in the efficient degradation of refractory organic pollutants.^56^ As shown in Figure 6a, it was obvious that MnO_*x*_/0.5C/SiO_2_ was the most efficient catalyzer to catalyze ozone decomposition, followed by MnO_*x*_/SiO_2_ and then ozone self-decomposition. The high decomposition of ozone could be attributed to the increased hydrophobicity, conductivity, and reducibility of MnO_*x*_/0.5C/SiO_2_. The adsorption of ozone with a low dipole moment of 0.46*D* was promoted, whereas, with good conductivity and reducibility, the activation of O_3_ was favored via electron transfer from the low-valent Mn species to ozone.

In situ diffuse reflectance infrared Fourier transform spectroscopy (in situ DRIFTS) was used to analyze the adsorption and decomposition of ozone on the surface of the catalysts. As shown in Figure 6b, several characteristic peaks appeared after O_3_ adsorption over MnO_*x*_/SiO_2_. The strong signal peaks at 1024–1054 cm^–1^ and the weak signal peaks at 2095–2122 cm^–1^ were attributed to chemically and physically adsorbed O_3_,^61^ respectively. The peaks at 1381 and 798 cm^–1^, corresponding to atomic oxygen (*O) and ozonide ions (O_3_^–^),^61^ also appeared. In contrast, these characteristic peaks of O_3_ adsorption and decomposition over MnO_*x*_/0.5C/SiO_2_ became much stronger (Figure 6c). Especially, the intensities of *O and O_3_^–^ peaks were strengthened with the time extension of ozone purging. The in situ DRIFTS results indicated that O_3_ was favored to be dissociated to form *O after its adsorption on the surface of MnO_*x*_/0.5C/SiO_2_. The active *O with high oxidation potential (2.43 V) endows MnO_*x*_/0.5C/SiO_2_ with excellent catalytic activity through its subsequent conversion into other powerful ROS.^62^

**Figure 7:**
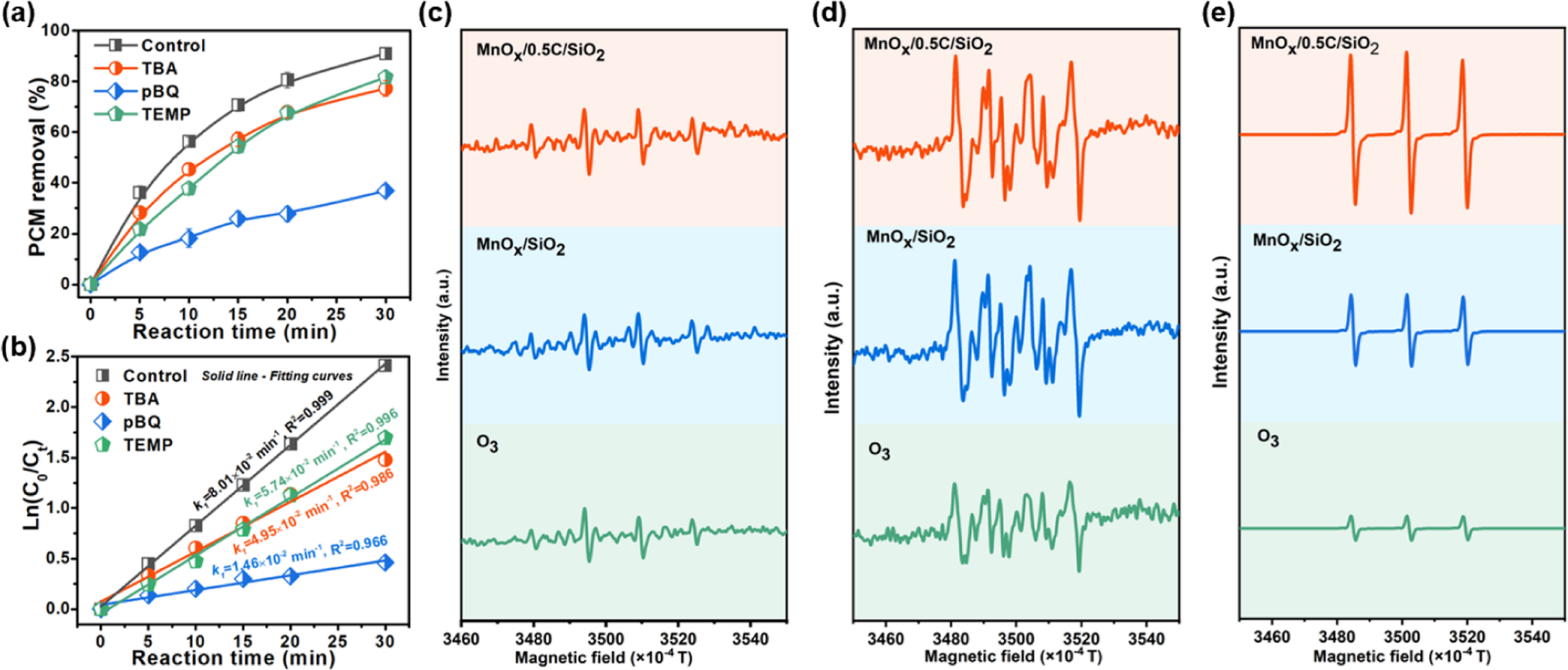
(a) PCM removal and (b) pseudo-first-order kinetics fitting in the MnO_*x*_/0.5C/SiO_2_-mediated catalytic ozonation with different scavengers. ESR spectra of (c) DMPO-^•^OH, (d) DMPO/DMSO-^•^O_2_^–^, and (e) TEMP–^1^O_2_ adducts in single ozonation and catalytic ozonation with the MnO_*x*_/SiO_2_ and MnO_*x*_/0.5C/SiO_2_ catalysts.

Quenching experiments were performed to identify the primary ROS participating in MnO_*x*_/0.5C/SiO_2_-catalyzed ozonation. Three scavengers, including *tert*-butanol (TBA, ^•^OH scavenger), *p*-benzoquinone (pBQ, ^•^O_2_^–^ scavenger), and 2,2,6,6-tetramethylpiperidine (TEMP, ^1^O_2_ scavenger),^63^ were added into the PCM-containing oxidation system to identify ^•^OH, ^•^O_2_^–^, and ^1^O_2_, respectively. As shown in Figure 7a, the PCM removal was decreased from 91.1 ± 2.4 to 77.2 ± 3.1%, 81.7 ± 2.3, and 36.9 ± 2.1% with the quenching of TBA, TEMP, and pBQ, respectively. The pseudo-first-order kinetic constant of PCM degradation was also suppressed by the three scavengers. As depicted in Figure 7b, MnO_*x*_/0.5C/SiO_2_ had a relatively low reaction rate constant of 5.74 × 10^–2^, 4.95 × 10^–2^, and 1.46 × 10^–2^ min^–1^ with the addition of TBA, TEMP, and pBQ, which were 0.72, 0.62, and 0.18 times that of (8.01 × 10^–2^ min^–1^) without any scavengers, respectively. Obviously, pBQ greatly suppressed the catalytic activity, suggesting that ^•^O_2_^–^ was the dominant active species in the MnO_*x*_/0.5C/SiO_2_-catalyzed ozonation, while ^1^O_2_ and ^•^OH were also involved. The ROS quantitative experiments (Figure S8a) further proved that the yield of ^•^O_2_^–^ reached as high as 77.2 μmol/L within 30 min in the MnO_*x*_/0.5C/SiO_2_ + O_3_ system, which was much higher than that (46.8 μmol/L) in the O_3_-alone system. In particular, the detected concentration of ^•^O_2_^–^ increased dramatically to 55.7 μmol/L within only 5 min and then gradually increased within 30 min, which might be due to the subsequent conversion of ^•^O_2_^–^ into other species during the chain reactions.

The presence of ROS in O_3_-alone, MnO_*x*_/SiO_2_ + O_3_, and MnO_*x*_/0.5C/SiO_2_ + O_3_ systems were compared in terms of the relative intensity of ESR signals. As shown in Figure 7c, with the addition of a DMPO trapping agent, the ESR signals of DMPO-^•^OH (quadruple peaks with an intensity ratio of 1:2:2:1) were observed in all three systems. The ^•^OH signal intensity was hardly enhanced after the introduction of MnO_*x*_/SiO_2_ and MnO_*x*_/0.5C/SiO_2_ into the O_3_-alone system, which was consistent with the ^•^OH quantitative results (Figure S8b). This further suggested that ^•^OH had little effect on the catalytic performance. On the contrary, the DMSO-^•^O_2_^–^ signal (quadruple peaks with an intensity ratio of 1:1:1:1) in the three systems was very strong and followed the order of MnO_*x*_/0.5C/SiO_2_ + O_3_ > MnO_*x*_/SiO_2_ + O_3_ > O_3_, implying that a large amount of ^•^O_2_^–^ was produced in the MnO_*x*_/0.5C/SiO_2_ catalytic ozonation system (Figure 7d). This was in good agreement with the ^•^O_2_^–^ quantitative results, that the yield of ^•^O_2_^–^ in the MnO_*x*_/0.5C/SiO_2_ + O_3_ system was 1.6 times higher than that of the O_3_-alone system (Figure S8a). It was observed that the TEMP–^1^O_2_ signal (triplet peaks with an intensity ratio of 1:1:1) also followed the order MnO_*x*_/0.5C/SiO_2_ + O_3_ > MnO_*x*_/SiO_2_ + O_3_ > O_3_ (Figure 7e). According to Ma’s report, the strong MSI effect inhibits the generation of ^1^O_2_ in catalytic ozonation.^19^ It could be speculated that the weak MSI induced by carbon modification was the decisive factor for the great enhancement of ^1^O_2_ generation in MnO_*x*_/0.5C/SiO_2_-catalyzed ozonation. Hence, the ESR results confirmed again that the weakened MSI effect through carbon modification promoted ozone activation to produce more ROS, thus improving the degradation of PCM.

#### DFT Calculation

**Figure 8:**
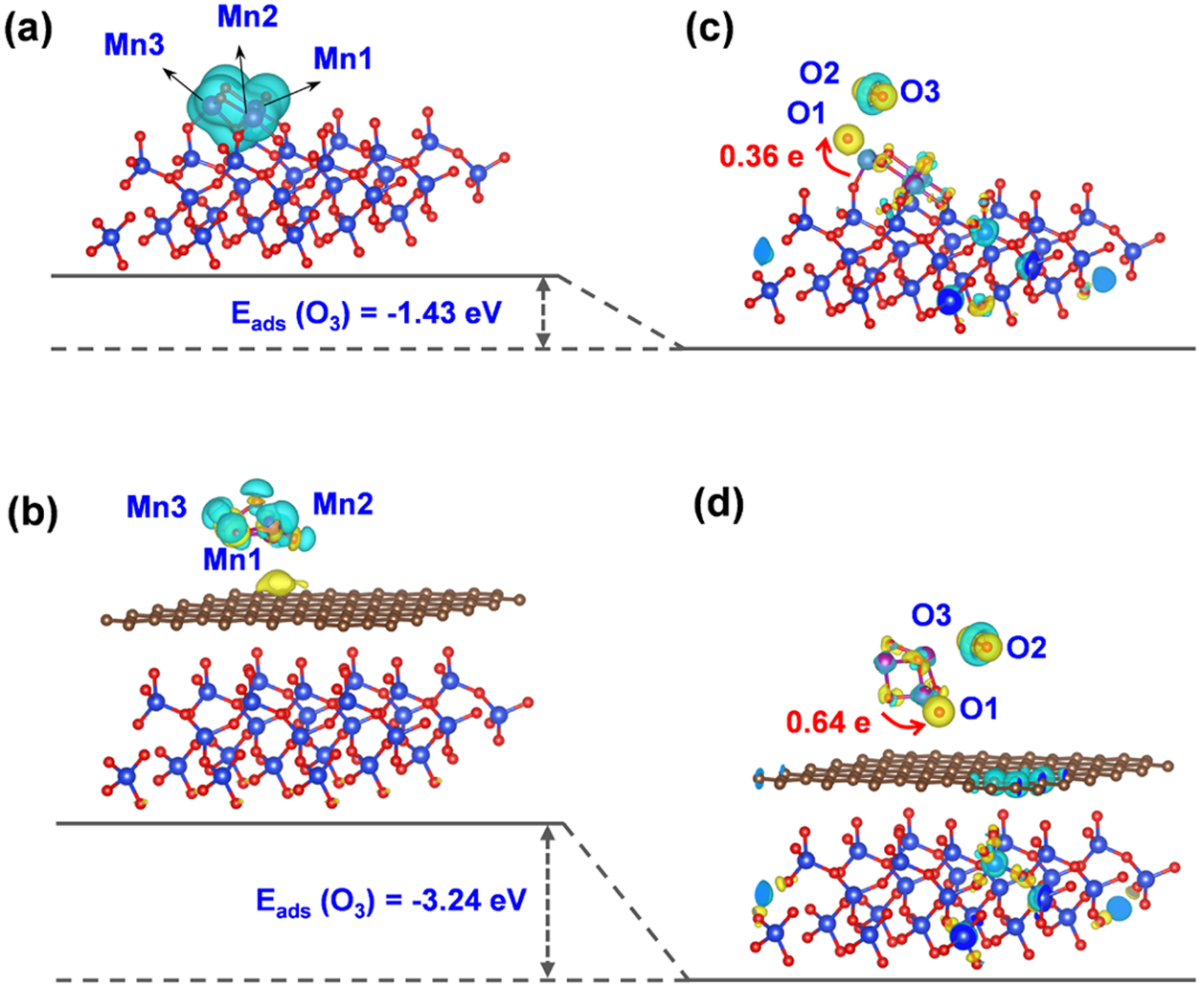
Charge-difference isosurfaces of (a) MnO_*x*_/SiO_2_ (side view) and (b) MnO_*x*_/0.5C/SiO_2_ (side view) before the adsorption of O_3_. Charge-difference isosurfaces of (c) MnO_*x*_/SiO_2_ (side view) and (d) MnO_*x*_/0.5C/SiO_2_ (side view) after the adsorption of O_3_.

DFT calculations were used to explore the transfer of electrons from Mn to Si via the Si–O–Mn bond. Figure S9 shows the optimized calculation models of MnO_*x*_/SiO_2_ and MnO_*x*_/0.5C/SiO_2_ before and after the adsorption of O_3_. The charge difference illustrated the charge transfer between Mn and Si in MnO_*x*_/SiO_2_ (Figures 8a and S10a) and MnO_*x*_/0.5C/SiO_2_ (Figures 8b and S10b), respectively. The blue and yellow isosurfaces depicted charge depletion and accumulation in the space, respectively. As shown in Figure 8a, the electrons of Mn1, Mn2, and Mn3 in the MnO_*x*_/SiO_2_ configuration were observed to lose 1.74, 1.68, and 1.70e, respectively. In contrast, the electrons of Mn1, Mn2, and Mn3 in the MnO_*x*_/0.5C/SiO_2_ configuration exhibited a loss of 1.28 e, 1.43, and 1.43 e, respectively (Figure 8b). The results indicated that the introduction of carbon film could prevent the electron transfer from Mn to Si due to the weak MSI effect between MnO_*x*_ and the SiO_2_ support. Subsequently, the adsorption energies (*E*_ads_) for the adsorption of O_3_ adsorbed on MnO_*x*_/SiO_2_ and MnO_*x*_/0.5C/SiO_2_ configuration structures were calculated. As shown in Figures 8c,d and S10c,d, the *E*_ads_ value for the adsorbed O_3_ on MnO_*x*_/SiO_2_ was −1.43 eV, whereas that on MnO_*x*_/0.5C/SiO_2_ was −3.24 eV, respectively, indicating the stronger O_3_ adsorption ability than MnO_*x*_/0.5C/SiO_2_. In addition, for the adsorption of O_3_ on MnO_*x*_/0.5C/SiO_2_, O_3_ gained charges of 0.64 e from the MnO_*x*_/0.5C/SiO_2_ catalyst, which was much higher than that from MnO_*x*_/SiO_2_ (0.36 e). These differences in charge density suggested that carbon-modified MnO_*x*_/0.5C/SiO_2_ more favored the adsorption and activation of O_3_ than MnO_*x*_/SiO_2_. The above experimental and theoretical calculation results prove that the carbonization of SiO_2_ anchored with MnO_*x*_ can achieve a weak MSI and reduce the electron transfer from Mn to Si. This allows the Mn site to remain electronegative, which is conducive to the adsorption and decomposition of electron-poor O_3_ molecules.

## Conclusions

In this study, MnO_*x*_ nanoclusters with a tiny size of 2.3–4.4 nm supported on carbon-coated silica (MnO_*x*_/*x*C/SiO_2_) were successfully synthesized. The metal–support interactions (MSIs) between MnO_*x*_ and SiO_2_ were tuned by simply introducing an intermediate carbon film. It was found that MnO_*x*_/0.5C/SiO_2_ with moderate MSI showed superior catalytic activity to the bare MnO_*x*_/SiO_2_ and the overmodified MnO_*x*_/1C/SiO_2_. Moreover, MnO_*x*_/0.5C/SiO_2_ also performed well in a wide pH range and under various realistic water scenarios, demonstrating its enormous potential for practical application in water and wastewater decontamination. The introduction of moderate carbon film weakened the MSI between MnO_*x*_ and SiO_2_ and thus increased the low-valent Mn abundance and redox properties of MnO_*x*_/*x*C/SiO_2_, which greatly facilitated the adsorption and activation of ozone. Moreover, the carbon modification also enhanced the conductivity and hydrophobicity of MnO_*x*_/0.5C/SiO_2_, which strengthened the mass transportation of electrons and organic pollutants. All of these characteristics derived from carbon modification contributed to the high catalytic activity of MnO_*x*_/0.5C/SiO_2_.

## Supporting Information Available

Characterization details; details of catalytic ozonation procedures in batch reactors and continuous-flow membrane reactor; details of computational methods; differential charge density maps of O_3_; TGA curves of 0.5C/SiO_2_ and 1C/SiO_2_; AC-HAADF-STEM images of the MnO_*x*_/0.5C/SiO_2_ catalyst; contact angle tests of SiO_2_, MnO_*x*_/SiO_2_, 0.5C/SiO_2_, MnO_*x*_/0.5C/SiO_2_, 1C/SiO_2_, and MnO_*x*_/1C/SiO_2_; PCM removal in adsorption with SiO_2_, 0.5C/SiO_2_, and 1C/SiO_2_; removal of PCM in real water matrix using MnO_*x*_/0.5C/SiO_2_ powder catalyst mediated catalytic ozonation; the stability test of MnO_*x*_/0.5C/SiO_2_ in the degradation of PCM; time profile of cumulative concentration of superoxide radicals and hydroxyl radicals in ozonation and catalytic ozonation; DFT calculation models of MnO_*x*_/SiO_2_ and MnO_*x*_/0.5C/SiO_2_ before the adsorption of O_3_; calculation models of MnO_*x*_/SiO_2_ and MnO_*x*_/0.5C/SiO_2_ after the adsorption of O_3_; charge-difference isosurfaces of MnO_*x*_/SiO_2_ (top view) and MnO_*x*_/0.5C/SiO_2_ (top view) before the adsorption of O_3_; charge-difference isosurfaces of MnO_*x*_/SiO_2_ (top view) and MnO_*x*_/0.5C/SiO_2_ (top view) after the adsorption of O_3_; TGA and XPS results of the catalysts; and parameters of tested water (PDF)
