# Supplementary material for: Weakening the Mn–O–Si Interaction via Carbon Intercalation for the Enhanced Catalytic Ozonation of Refractory Pollutants in Environmental Matrices
Source: ACS Appl Mater Interfaces. 2025 Feb 17;17(8):12177–88. doi: 10.1021/acsami.4c21068 (PMC11874039; doi:10.1021/acsami.4c21068)
Supplement: Supplementary file 1 — am4c21068_si_001.pdf [file am4c21068_si_001.pdf]

Supporting Information

for

Weakening the Mn-O-Si Interaction via Carbon Intercalation for Enhanced Catalytic Ozonation of Refractory Pollutants in Environmental Matrices

Huating Huang [a], Weiqing Li [a], Xixi Chen [c,*], Zhiming Yang [d], Minggang Chen [d], Anhong Zhang [d], Chun He [a,b], Shuanghong Tian [a,b,*]

[a] School of Environmental Science & Engineering, Sun Yat-sen University, Guangzhou 510275, P. R. China

[b] Guangdong Provincial Key Laboratory of Environmental Pollution Control and Remediation, Guangzhou 510275, P. R. China

[c] Department of Materials Science and Engineering, City University of Hong Kong, P. R. China

[d] China National Chemical Southern Construction Investment Co., Ltd., Guangzhou 516000, P.R. China

* Corresponding author: Tel.: +86 20 84115556; fax: +86 20 39332690.

E-mail address: xixichen@cityu.edu.hk, chenxixi8117@163.com (Xixi Chen); tshuangh@mail.sysu.edu.cn (Shuanghong Tian)

Contents

Text S1. Characterization details.

Text S2. Details of catalytic ozonation procedures in batch reactors and continuous-flow membrane reactor.

Text S3. Details of computational methods.

Fig. S1. Differential charge density maps of O_3.

Fig. S2. TGA curves of $0.5C/SiO_2$ and $1C/SiO_2$.

Fig. S3. AC-HAADF-STEM images of $MnO_x/0.5C/SiO_2$ catalyst.

Fig. S4. Contact angle tests of (a) SiO_2, (b) MnO_x/SiO_2, (c) $0.5C/SiO_2$, (d) $MnO_x/0.5C/SiO_2$, (e) $1C/SiO_2$ and (f) $MnO_x/1C/SiO_2$

Fig. S5. PCM removal in adsorption with SiO_2, $0.5C/SiO_2$ and $1C/SiO_2$.

Fig. S6. Removal of PCM in real water matrix using $MnO_x/0.5C/SiO_2$ powder catalyst mediated catalytic ozonation.

Fig. S7. The stability test of $MnO_x/0.5C/SiO_2$ in the degradation of PCM.

Fig. S8. Time profile of cumulative concentration of (a) superoxide radicals and (b) hydroxyl radicals in ozonation and catalytic ozonation.

Fig. S9. DFT calculation models of (a) MnO_x/SiO_2 and (b) $MnO_x/0.5C/SiO_2$ before adsorption of O_3. Calculation models of (c) MnO_x/SiO_2 and (d) $MnO_x/0.5C/SiO_2$ after adsorption of O_3.

Fig. S10. Charge difference iso-surfaces of (a) MnO_x/SiO_2 (top view) and (b) $MnO_x/0.5C/SiO_2$ (top view) before adsorption of O_3. Charge difference iso-surfaces of (c) MnO_x/SiO_2 (top view) and (d) $MnO_x/0.5C/SiO_2$ (top view) after adsorption of O_3.

Table S1. TGA and XPS results of the catalysts.

Table S2. Parameters of tested water.

Text S1. Characterization details.

The carbon content and thermal stability of the samples were examined using a thermogravimetric analyzer (TGA, TG209F1 libra, NETZSCH, Germany) in an air atmosphere. The morphologies of the samples were characterized by scanning electron microscopy (SEM, Gemini SEM500, Zessi), a high-resolution transmission electron microscopy (HRTEM, Tecnai G2 F30, FEI, USA) with an acceleration voltage of 300 kV and energy dispersive X-ray (EDS) detector, and a spherical-aberration-corrected transmission electron microscopy (AC-TEM, JEM-ARM 200P, JEOL, Japan). Their crystal structure and phase composition were measured by X-ray diffraction (XRD, Ultima IV, Rigaku Co., Japan) with the Cu Kα radiation at 40 kV and 40 mA. Laser confocal Raman spectroscopy (inVia Qontor, Renishaw plc, UK) and Fourier transform infrared spectroscopy (FTIR, Nicolet iS50, Thermo Fisher, USA) were used to analyze the substance composition and the chemical bonds of catalysts. Surface elemental analysis of the samples was performed using X-ray photoelectron spectroscopy (XPS, Thermo Scientific NEXSA, ThermoFisher, USA). Electron paramagnetic resonance (ESR, Bruker EMXplus, Germany) was conducted for identifying the oxidant states of solid metal oxides and reactive oxygen species (ROS) in solution. The electron transfer and redox capacity of the samples were analyzed by cyclic voltammetry (CV) using an electrochemical workstation (CHI 660E, Zahner, Germany). Autosorb-iQ (AutoChem II, Micromeritics, USA) was used to analyze the hydrogen temperature programmed reduction (H_2-TPR) of the samples.

Text S2. Details of catalytic ozonation procedures in batch reactors and continuous-flow membrane reactor.

For batch reaction tests, catalytic ozonation experiments were conducted in a quartz column reactor at a semi-batch mode. For each run, 100 mL of wastewater containing 40 mg/L PCM and 0.03 g catalysts were charged into the reactor under magnetic stirring and at 25 °C (regulated by a water bath). The adsorption of pollutants by the catalyst was determined after 30 min. Then, ozone with a concentration of 1.2 mg/L and a flow rate of 200 mL/min, generated by an ozone generator (YE-TG-01PII, Nanjing YDG ozone Co., Ltd., China), was injected into the reactor. The residue ozone from the system was quenched by passing it into a KI solution. At fixed intervals, 2.0 mL of sample was collected, immediately purged with N_2, and filtered through 0.22 μm filters. The initial pH of the solution during the above reaction was 6.3 and was not controlled any more during the reaction.

For continuous-flow membrane performance tests, catalytic ozonation reactions in MnO_x/0.5C/SiO_2-PVDF membrane were performed using a filtration setup in **Fig. 5a−b**. Firstly, the catalytic MnO_x/0.5C/SiO_2-PVDF membrane was fixed inside the reaction module and securely sealed with O-ring rubber bands to prevent water leakage. Subsequently, the feed solution (e.g., tap water, surface water and simulated aquaculture wastewater) was fed into the membrane module at a specified flow rate. Meanwhile, the ozone, produced from pure oxygen by the ozone generator, was continuously bubbled into the reactor at a constant flow rate. The permeate samples were taken at 30 min intervals, filtered with 0.22 μm filters for further analysis.

Text S3. Details of computational methods.

Calculations were performed based on density functional theory as implemented in the Vienna ab initio simulation package (VASP) code with the projector augmented plane-wave method [1-3]. The generalized gradient approximation (GGA) described by Perdew-Burke-Ernzerh (PBE) function was used for exchange-correlation potential [2]. To eliminate the error of electron self-interaction of localized Mn 3d-orbital, DFT+U method with a U value of 2.8 eV for the Mn 3d state were employed in the calculations [3]. The cutoff energy of 400 eV and the Brillouin zone of a 3×3×1 k-points mesh were used for geometry optimization. The convergence of energy criterion and force of all the relaxed atoms was set as 10^{-5} eV and 0.02 eV/Å, respectively.

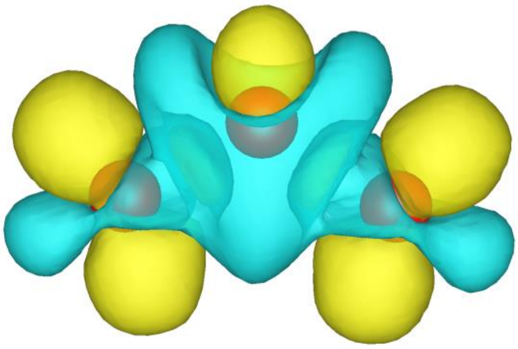

Fig. S1. Differential charge density maps of O_3.

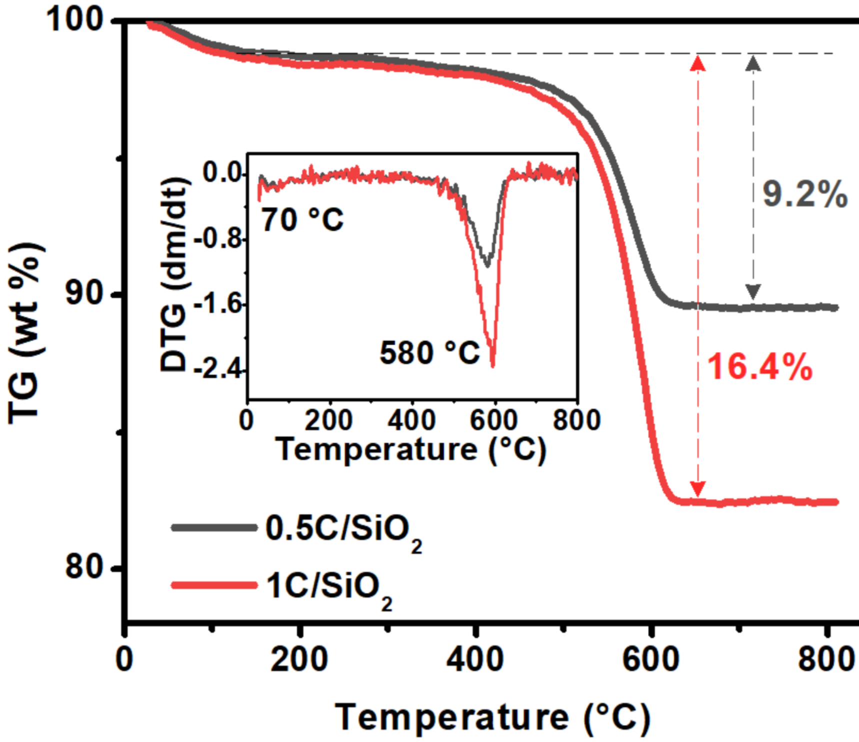

Fig. S2. TGA curves of 0.5C/SiO_2 and 1C/SiO_2.

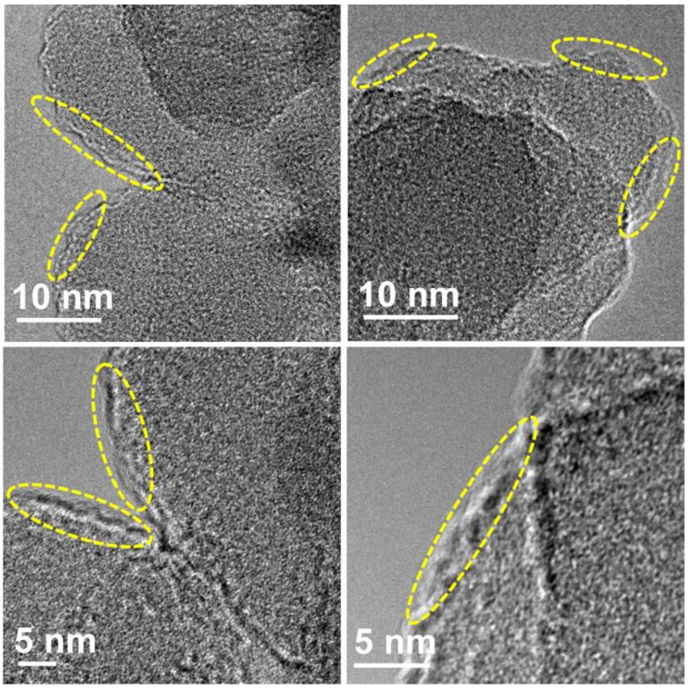

Fig. S3. AC-HAADF-STEM images of MnO_x/0.5C/SiO_2 catalyst.

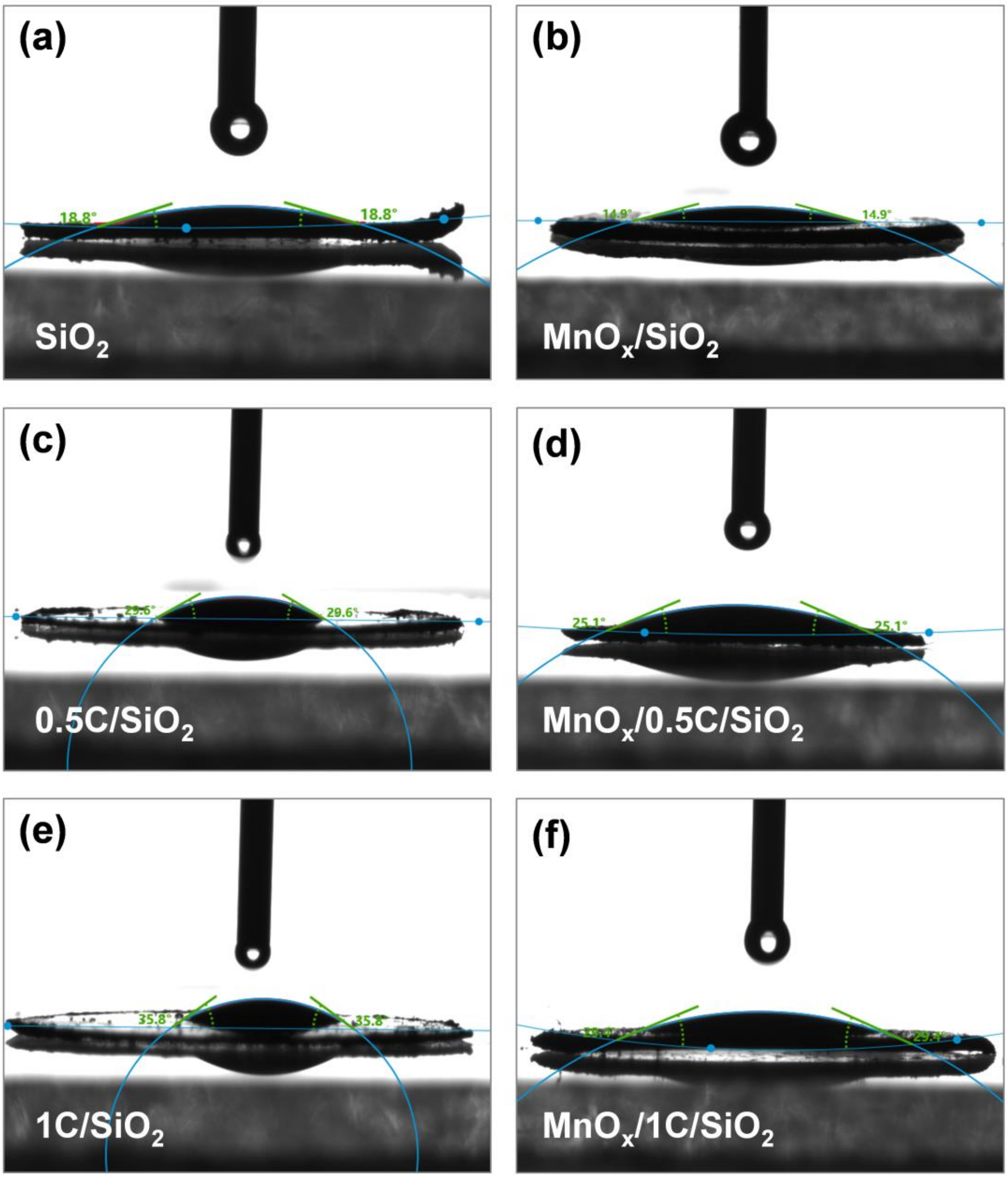

Fig. S4. Contact angle tests of (a) SiO_2, (b) MnO_x/SiO_2, (c) $0.5C/SiO_2$, (d) $MnO_x/0.5C/SiO_2$, (e) $1C/SiO_2$ and (f) $MnO_x/1C/SiO_2$.

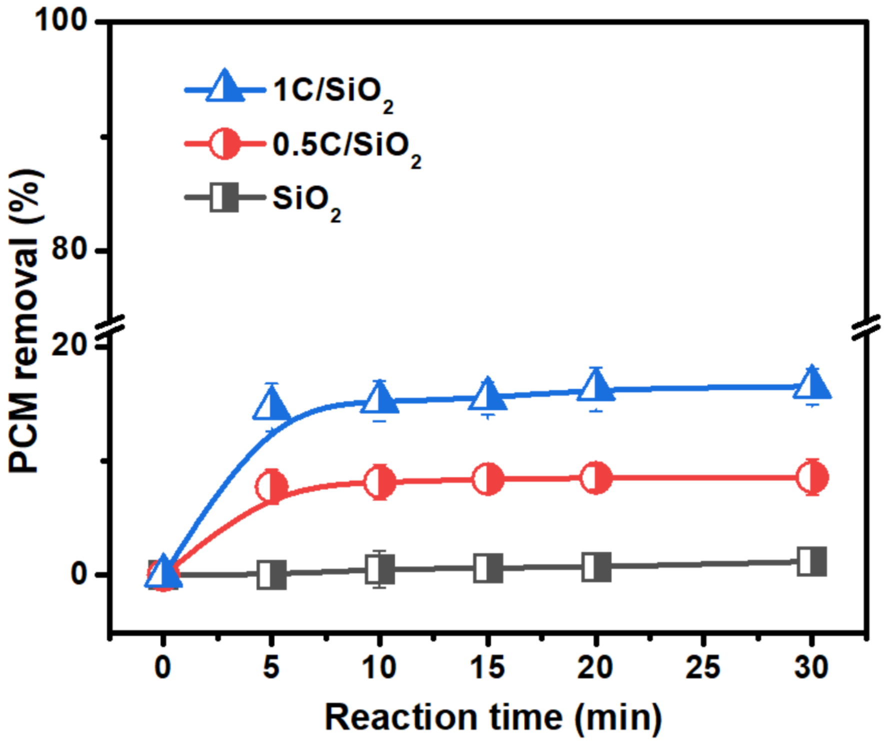

Fig. S5. PCM removal in adsorption with SiO_2, $0.5C/SiO_2$ and $1C/SiO_2$.

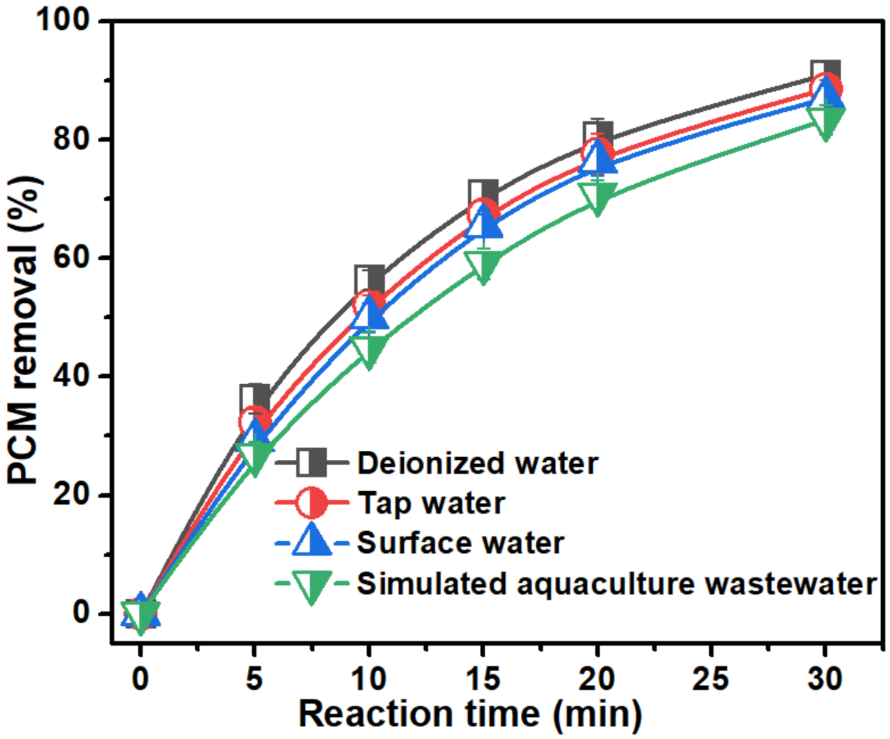

Fig. S6. Removal of PCM in real water matrix using MnO_x/0.5C/SiO_2 powder catalyst mediated catalytic ozonation.

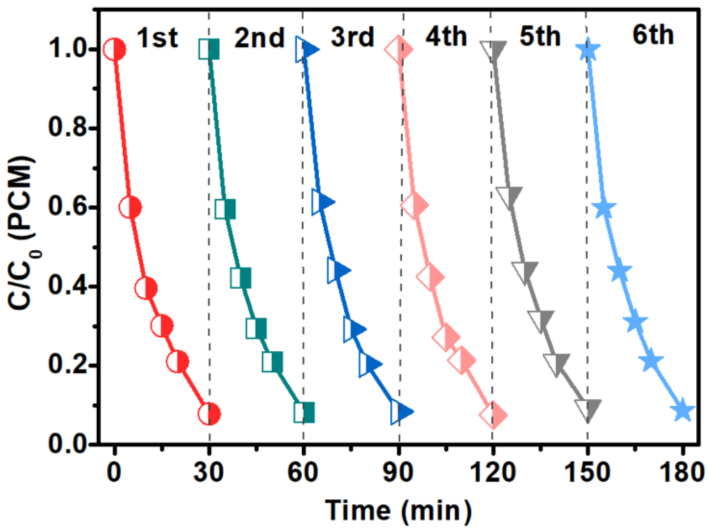

Fig. S7. The stability test of MnO_x/0.5C/SiO_2 in the degradation of PCM.

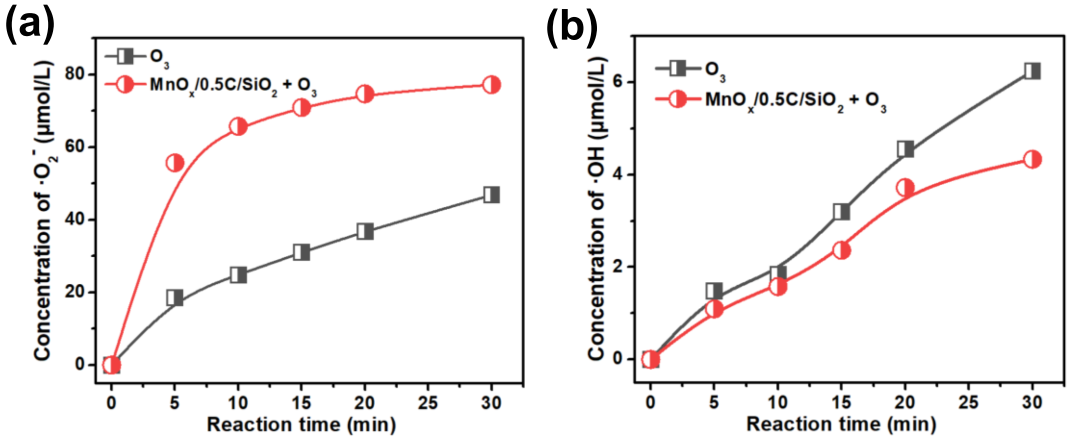

Fig. S8. Time profile of cumulative concentration of (a) superoxide radicals and (b) hydroxyl radicals in ozonation and catalytic ozonation.

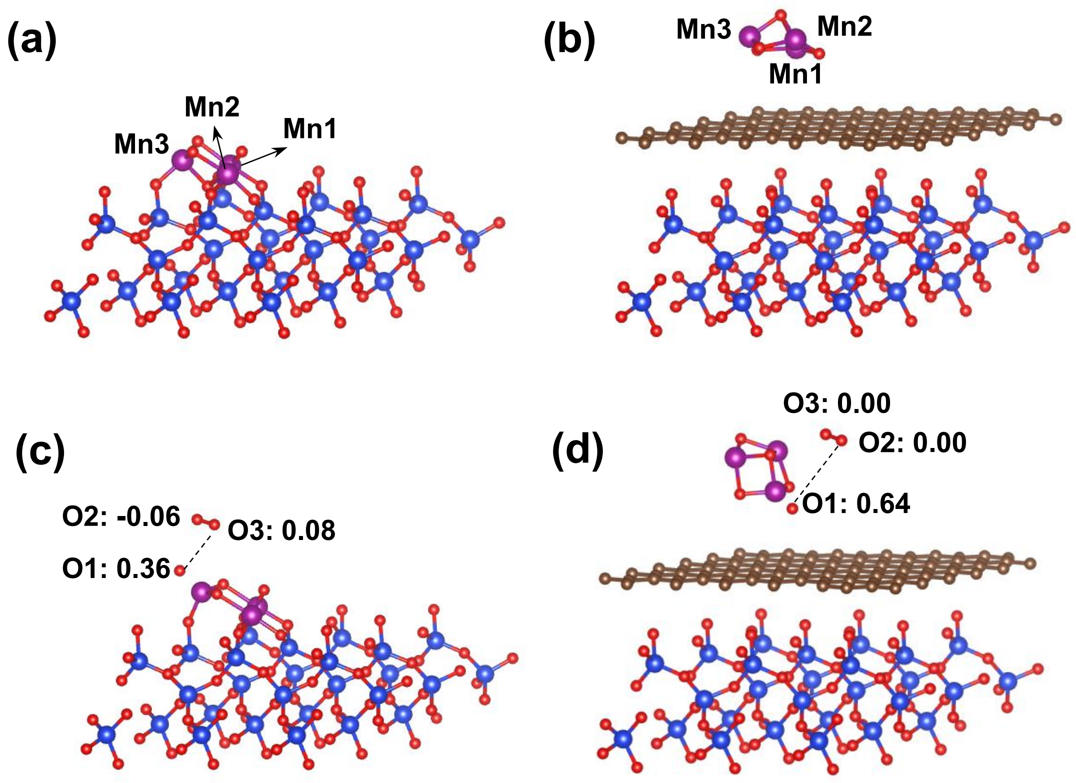

Fig. S9. DFT calculation models of (a) MnO_x/SiO_2 and (b) $MnO_x/0.5C/SiO_2$ before adsorption of O_3. Calculation models of (c) MnO_x/SiO_2 and (d) $MnO_x/0.5C/SiO_2$ after adsorption of O_3.

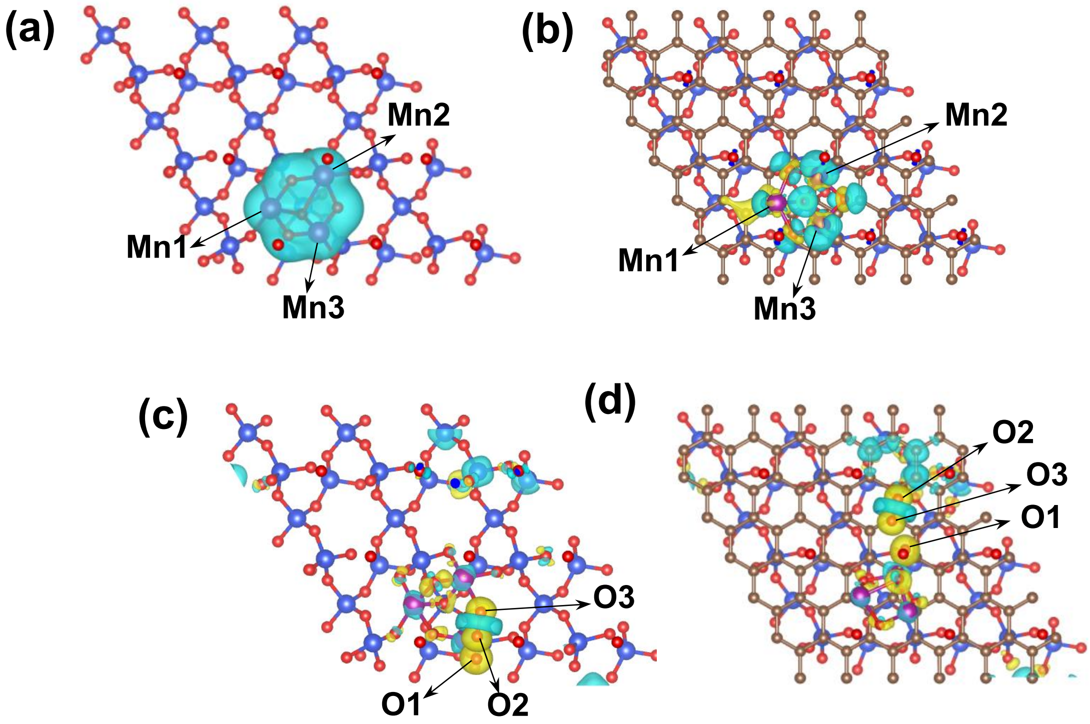

Fig. S10. Charge difference iso-surfaces of (a) MnO_x/SiO_2 (top view) and (b) $MnO_x/0.5C/SiO_2$ (top view) before adsorption of O_3. Charge difference iso-surfaces of (c) MnO_x/SiO_2 (top view) and (d) $MnO_x/0.5C/SiO_2$ (top view) after adsorption of O_3.

Table S1. TGA and XPS results of the catalysts.

Samples	C (wt %)	XPS				
		O_{ads}/O_{total} (%)	O_{surf}/O_{total} (%)	$[Mn^{2+}]/[Mn_{total}]$ (%)	$([Mn^{2+}]+[Mn^{3+}])/[Mn_{total}]$ (%)	AOS_{Mn}
MnO_x/SiO_2	0	58.5	36.7	31.9	64.3	2.54
$MnO_x/0.5C/SiO_2$	7.5	51.2	42.3	36.1	71.1	2.20
$MnO_x/1C/SiO_2$	15.1	26.7	58.1	37.6	67.6	2.31

Note: $[Mn_{total}]$ means the content of $([Mn^{2+}]+[Mn^{3+}]+[Mn^{4+}])$.

Table S2. Parameters of tested water.

Parameters	Deionized water	Tap water	Surface water	Simulated aquaculture wastewater
pH	5.95	7.02	7.60	8.21
COD_{Mn} (mg/L)	/	1.14	2.40	2.65
TOC (mg/L)	/	1.15	2.25	2.32
UV_{254} (cm^{-1})	/	0.01	0.05	0.08
Turbidity	/	0.11	4.46	7.58
DO (mg/L)	8.12	7.40	7.01	7.77

Note: the surface water was collected from a local drinking water treatment plant (Shenzhen, China).

References

1. Kresse, G.; Furthmuller, J. Efficiency of Ab-Initio Total Energy Calculations for Metals and Semiconductors Using a Plane-Wave Basis Set. *Comp. Mater. Sci.* **1996,** *6* (1), 15–50.

2. Perdew, J. P.; Burke, K.; Ernzerhof, M. Generalized Gradient Approximation Made Simple. *Phys. Rev. Lett.* **1996,** *77* (18), 3865–3868.

3. Rong, S.; Zhang, P.; Liu, F.; Yang, Y. Engineering Crystal Facet of Alpha-MnO_2 Nanowire for Highly Efficient Catalytic Oxidation of Carcinogenic Airborne Formaldehyde. *ACS Catal.* **2018,** *8* (4), 3435-3446.
